# Metabolic Reprogramming in Colon Cancer Cells Persistently Infected with Newcastle Disease Virus

**DOI:** 10.3390/cancers15030811

**Published:** 2023-01-28

**Authors:** Tong Yu, Archana Chandrabhan Jadhav, Jiabao Xu, Adrian L. Harris, Venugopal Nair, Wei E. Huang

**Affiliations:** 1 Department of Engineering Science, University of Oxford, Parks Road, Oxford OX1 3PJ, UK; 2 Beamline B24, Diamond Light Source, Harwell Science and Innovation Campus, Oxfordshire OX11 0DE, UK; 3 Viral Oncogenesis Group, The Pirbright Institute, Surrey GU24 0NF, UK; 4 Molecular Oncology Laboratories, Department of Oncology, Weatherall Institute of Molecular Medicine, John Radcliffe Hospital, Oxford University, Oxford OX3 9DS, UK; 5 Department of Biology, University of Oxford, 11a Mansfield Road, Oxford OX1 3SZ, UK

**Keywords:** Newcastle disease virus, Raman spectroscopy, cancer resistance, drug resistance, single cells

## Abstract

**Simple Summary:**

It is vitally important to understand the development of cancer resistance to therapies. Newcastle disease virus (NDV) is a promising oncolytic agent for cancer therapy. A small subpopulation of Caco-2 colon cancer cells persistently infected with NDV was found, which demonstrated resistance to NDV reinfection. By applying Raman spectroscopic and stable isotopic techniques, we found possible mechanisms of the resistant cells to escape from the viral attack, by slowing down their replication and diverting the energy to protein and lipid synthesis. Understanding metabolic reprogramming would be extremely helpful in creating novel cancer treatments to identify and target resistant cells at the single-cell level with great precision.

**Abstract:**

Newcastle disease virus (NDV) is an oncolytic agent against various types of mammalian cancers. As with all cancer therapies, the development of cancer resistance, both innate and acquired, is becoming a challenge. In this study, we investigated persistently NDV-infected Caco-2 colon cancer cells, designated as virus-resistant (VR) Caco-2 cells, which were then able to resist NDV-mediated oncolysis. We applied single-cell Raman spectroscopy, combined with deuterium isotope probing (Raman-DIP) techniques, to investigate the metabolic adaptations and dynamics in VR Caco-2 cells. A linear discriminant analysis (LDA) model demonstrated excellent performance in differentiating VR Caco-2 from Caco-2 cells at single-cell level. By comparing the metabolic profiles in a time-resolved manner, the de novo synthesis of proteins and lipids was found upregulated, along with decreased DNA synthesis in VR Caco-2. The results suggest that VR Caco-2 cells might reprogram their metabolism and divert energy from proliferation to protein synthesis and lipidic modulation. The ability to identify and characterise single resistant cells among a population of cancer cells would help develop a deeper understanding of the resistance mechanisms and better tactics for developing effective cancer treatment.

## 1. Introduction

Newcastle disease virus (NDV) is a single-stranded RNA avulavirus causing Newcastle disease. Over the last five decades, NDV has been known to have oncolytic properties and is a potential candidate for oncovirotherapy due to its tumour-selective replication sparing healthy cells [1]. Tumours provide a favourable environment for NDV replication, spreading due to compromised antiviral defences related to the weaker sensitivity and expressions of type 1 interferon (IFN) as well as developing resistance to cell death pathways [2]. Since 1957, when NDV’s anti-tumour potential was first demonstrated in NDV-coated ascitic cancer cells [3], both preclinical and clinical research have made substantial use of NDV as an oncolytic drug, with its non-pathogenic strains (e.g., Hitchner B1, LaSota, Ulster and V4UPM), mildly pathogenic strains (e.g., PV701 and 73T) and highly pathogenic strains (e.g., AF2240) all proven to be potent anti-cancer agents.

Despite promising responses observed in clinical studies, persistent NDV infections have been reported in a range of in vitro cancer cell infections [4,5,6]. In colorectal cancer, the persistently infected cells were discovered to harbour viral offspring that formed smaller plaques, in contrast to the uninfected cancer cells [4]. Hyperfusogenic NDV activity and the emergence of persistent infection were linked by two alterations in the glycoproteins of NDV: F and HN proteins in persistently infected ovarian cancer [5]. Upregulation of genes associated with cell growth, pro-survival and anti-apoptosis have also been reported during the development of persistently infected bladder cancer cells [6]. Persistent NDV infections in cancer cells result in reduced NDV-induced oncolysis and therefore hinder the development and translation of NDV as an oncolytic virotherapy. As the mechanism of persistent NDV infection in cancer cells remains poorly understood, further investigation and identification of the persistency in cancer cells is critical.

Here, we apply label-free, single-cell Raman spectroscopy for investigating cellular resistance at single-cell level. This spectroscopic technique can provide a comprehensive “biomolecular fingerprint” of a biological system [7]. In combination with advanced confocal microscopy, the biomolecular fingerprint of a single cell can be obtained as a single-cell Raman spectrum (SCRS) [8]. In addition, Raman techniques can be integrated with deuterium isotope probing (DIP) to map the general metabolic activity of a cell [9]. By incubating cells with heavy water (D_2_O), the D atoms in D_2_O can exchange with H atoms and form a variety of C–D bonds mediated by NADPH. By quantifying the C–D band at 2170–2300 cm^−1^ in the Raman spectrum, the degree of D incorporation can be used to estimate the anabolic activity. This method has been widely used to research the ecological and microbiological activity of microbes [9,10,11,12]. Recently, Raman-DIP using D_2_O has also been reported as a way of studying the metabolism of cancer cells [13,14]. Raman-DIP has many advantages over other targeted probes, for example, fluorophores, that are invasive and may interfere with a cell’s inherent metabolic activity.

In this study, we unintentionally observed the emergence of persistent infection in Caco-2 cells, initially the most sensitive to the NDV strain, after infection in a succession of cancer cell lines with a non-pathogenic strain of NDV, B1-GFP. The persistently infected cells were isolated and named virus-resistant (VR) Caco-2. We employed single-cell Raman spectroscopy and Raman-DIP to explore the metabolic differences within the VR Caco-2 cells and compared these to the parental uninfected Caco-2 cells. Raman spectral profiles of the VR Caco-2 cells were found to be distinctly different from those of the uninfected Caco-2 cells, suggesting significant metabolic reprogramming in those persistently infected cells.

## 2. Materials & Methods

### 2.1. Virus Strain and Cell Lines Used in This Study

The virus strain used in this study was a non-pathogenic strain of NDV Hitchner B1-GFP, obtained from the Department of Microbiology, Mount Sinai School of Medicine, New York.

Human cancer lines used include lung adenocarcinoma A549, hepatocellular Hep G2, prostate adenocarcinoma PC3, cervical adenocarcinoma HeLa, colorectal adenocarcinoma Caco-2 and virus-resistant Caco-2 (VR Caco-2). A non-cancer human lung fibroblast cell line MRC5 was also used. A549, Hep G2, HeLa, Caco-2, PC3 and MRC cell lines were obtained from the cell line inventory run by the Pirbright Institute’s cell services unit. A549, MRC5, PC3, Caco-2 and VR Caco-2 cells were authenticated using short tandem repeat (STR) profiling from ECACC.

For VR Caco-2 cells, Caco-2 cells were first infected with undiluted stock of B1-GFP virus, resulting in an estimated multiplicity of infection (MOI) of 50. About >98% cells were infected within 24 h and >95% of infected cells were dead within 48 h post-infection. A few cells survived 12 days post-infection and were identified by cultivation and further isolated as VR Caco-2 cells.

### 2.2. Cell Culture and Virus Infection

The complete media used for culturing each cell line are as follows. For culturing Caco-2 and VR Caco-2 cells, minimum essential medium eagle (MEM) with Earle’s salts and sodium bicarbonate without L-Glutamine (Sigma-Aldrich, Gillingham, Dorset, UK) was supplemented with 1.0 mg/mL D-glucose, 1% penicillin-streptomycin (10,000 U/mL), 1× NEAA, 4 mM L-glutamine, 1 μg/mL amphotericin B and 20% foetal bovine serum (FBS). For culturing Hep G2 and MRC cells, MEM was used, supplemented with 1.0 mg/mL of D-glucose, 1% penicillin-streptomycin, 4 mM L-glutamine, 1 μg/mL amphotericin B and 10% FBS. Roswell Park Memorial Institute (RPMI) (2.2 mg/mL of D-glucose) medium (Sigma-Aldrich) and Ham’s F-12K (Kaighn’s) (1.26 mg/mL of D-glucose) medium (Gibco, Thermo Fisher Scientific, Loughborough, UK) were used for culturing HeLa cells and PC3 cells, respectively, both supplemented with 1% penicillin-streptomycin, 1 μg/mL amphotericin B and 10% FBS. For culturing A549 cells, Dulbecco’s modified eagle’s medium (DMEM) with high glucose of 4.5 μg/mL, L-glutamine and sodium bicarbonate without sodium pyruvate (Sigma-Aldrich) was supplemented with 1% penicillin-streptomycin, 1 mM sodium pyruvate, 1 μg/mL amphotericin B and 10% FBS. The cell culture media was changed every 48 h during the prolonged cell-culturing process.

### 2.3. Cell Viability and Toxicity Assay

CellTiter-Blue^®^ Cell Viability Assay (Promega, Southampton, UK) was used to compare the viability of NDV-infected cells to mock-infected control cells. The manufacturer’s instructions were followed. The reduction reaction of the resorufin to the fluorescent product was done by incubating the metabolically active cells at 37 °C and 5% CO_2_ for up to 4–6 h. A Promega GloMax^®^ Explorer Multimode Microplate Reader was used to measure the fluorescence generated at an excitation of 560 nm and emission at 590 nm. The measured viability fraction was computed by dividing the normalised fluorescence reading of the infected cells by that of the uninfected cells.

CellToxTM Green Cytotoxicity Assay (Promega) was used to evaluate cell toxicity brought on by NDV infection. The DNA-binding cyanine dye was only taken up by dead cells due to a breakdown of cell membrane integrity and the fluorescent signal was detected at 520–530 nm. The manufacturer’s instructions were followed when performing the end-point cytotoxicity assay at day 4 of infection by B1-GFP (MOI~0.1) to Caco-2 and VR Caco-2 cells. The fold change in cytotoxicity was calculated by cytotoxicity of NDV-infected cells divided by cytotoxicity of mock-infected cells.

### 2.4. Cell Proliferation Assay

CellTrace^TM^ CFSE Cell Proliferation Kit (Invitrogen^TM^, Thermo Fisher Scientific, Loughborough, UK) and flow cytometry analysis of CFSE-labelled cells at 488 nm were used to measure cell proliferation in Caco-2 and VR Caco-2 cells. Cells were cultured in complete growth media at 37 °C inside a 5% CO_2_ incubator until 80–90% confluence was reached. Cells were then trypsinised with 0.25% trypsin-EDTA solution, centrifuged at 325× *g*, and washed and resuspended with serum-free MEM media. CFSE buffer was added to the resuspended cells to reach 1 × 10^6^ cells/mL in 2.5 μM CFSE buffer. The mixture was then incubated in a water bath at 37 °C for 15 min to label DNA. Following a serum-free MEM medium wash, CFSE-labelled cells were resuspended in complete growth media at 37 °C for 1 hour. After washing and resuspending, 5 × 10^5^ cells per well were seeded into 24-well plates, which were then cultured at 37 °C with 5% CO_2_. Using a MACSQuant Analyzer (Miltenyi Biotec, Bisley, Woking, UK), the fluorescence of the CFSE-labelled cells was monitored at five different time intervals (0, 12, 24, 48 and 72 h) after labelling. The Incucyte Live-Cell Analysis system (Sartorius, Epsom, UK) was used to monitor the proliferation of Caco-2 and VR Caco-2 cells seeded at different densities (5 k/well, 10 k/well and 15 k/well) in real time.

### 2.5. Caco-2 and VR Caco-2 Cells for Raman and Raman-DIP Measurements

Non-infected parental Caco-2 and VR Caco-2 cells were grown for up to 5–6 days until 80–90% confluence was attained. In Raman-DIP experiments, Caco-2 complete media with 30% D_2_O (Sigma-Aldrich) were used for culturing Caco-2 and VR Caco-2 cells, with cells obtained at different time points at 0, 12, 24, 48 and 72 h. The 30% D_2_O Caco-2 media was formulated by using 10 mL of 10× MEM (10 mg/mL of D-glucose) without L-glutamine and sodium bicarbonate (Sigma-Aldrich), 30 mL of 99% D_2_O (Sigma-Aldrich), 1 mL of 100× NEAA (Gibco), 1 mL of 200 mM L-glutamine (Gibco), 3 mL of 7.5% sodium bicarbonate solution (Sigma-Aldrich), 1 mL of penicillin-streptomycin (10,000 U/mL) (Gibco), 0.04 mL of Amphotericin B (Gibco), 20 mL of heat-inactivated FBS and 33.6 mL of sterile deionised water.

The cells were washed with PBSa and trypsinised. Trypsin was neutralised using serum-containing media and cells were washed with PBSa at 325 g for 5 min. Following that, cells were resuspended with cold solution of 4% paraformaldehyde (PFA) in PBSa and fixed for 1 hour at room temperature. The PFA-fixed cells were then washed once with PBSa and twice with Milli-Q water, following which the cell suspensions were dropped onto an aluminium-coated Raman slide and air dried before the measurements.

### 2.6. Single-Cell Raman Spectroscopic Measurements and Analysis

A LabRAM HR Evolution Raman microscope (Horiba Scientific, UK) was used for spectroscopic measurements, equipped with a 532-nm neodymium-yttrium aluminium garnet laser. Raman spectra were acquired via a 50× objective lens and 300 grooves/mm diffraction grating using 90-mW input laser power through a 25% neutral density (ND) filter and 3-s acquisition time. Raman scattering was detected by a charge-coupled device (CCD) cooled at −68 °C. The spectra were acquired in the range of 300 to 3400 cm^−1^. For VR Caco-2 and Caco-2 cells, 218 and 92 single cells were measured, respectively. In Raman-DIP experiments, >30 cells were measured for each time point. For each cell, around 30 spectra were measured. As the dried cells lost the original spatial distribution, spectra were averaged into one spectrum per single cell, which was used in the following analysis.

All spectra were pre-processed by cosmic ray correction and polyline baseline fitting using LabSpec 6 (Horiba Scientific, Montpellier, France). The entire spectrum region was vector normalised. Data analysis, statistics and visualisation were carried out in an R environment. Intracellular biomolecules were semi-quantified by quantifying corresponding band areas. The C–D (2070–2300 cm^−1^) and C–H (2800–3030 cm^−1^) band areas were quantified to determine deuterium content originating from incubation with D_2_O, while the metabolic rate was calculated by the ratio of (C–D) / (C–D) + (C–H). Performance of the statistics was done between single cells of VR Caco-2 and Caco-2 at each Raman wavenumber by comparing their intensities with Student’s *t*-test (ns: *p* > 0.05; *: *p* < 0.05; **: *p* < 0.01; ***: *p* < 0.001; ****: *p* < 0.0001).

## 3. Results & Discussion

### 3.1. Cell Line Screening for Establishing an NDV-Susceptible Cancer Cell System

**Figure 1:**
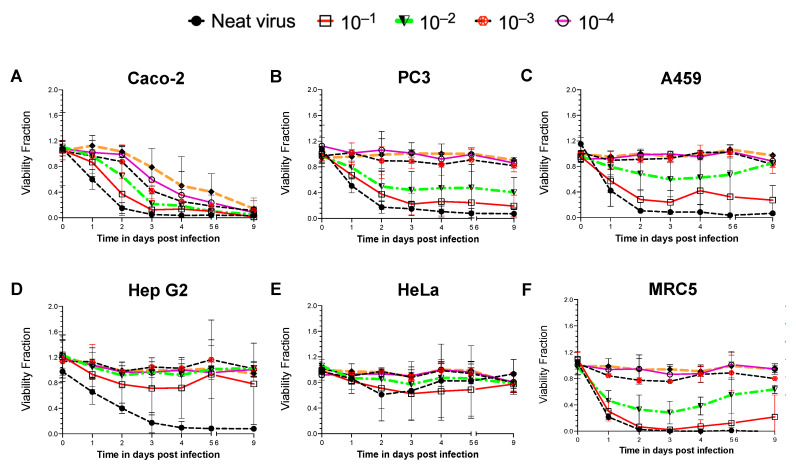
Time- and NDV dose-dependent cell viability assay in different human cancer cells. Cell lines were infected with a neat virus (MOI of 50) of a non-pathogenic reporter strain of NDV B1-GFP and its 10-fold dilutions. Cell lines derived from different cancerous tissues such as (**A**) the colon (Caco-2), (**B**) prostate adenocarcinoma cells derived from the metastatic site in bone (PC3), (**C**) lung (A549), (**D**) liver (Hep G2), (**E**) cervix (HeLa) and (**F**) non-cancerous human lung fibroblast cells (MRC5) were used for the study. Cell viability was measured throughout nine days of infection study. Data (mean ± SD) represented here is the combination of three repeats for each cell line. Viability Fraction = Normalised fluorescence reading of infected cells at (560 Ex/590 Em)/Normalised fluorescence reading of uninfected cells at (560 Ex/590 Em).

A non-pathogenic reporter strain of NDV, Hitchner B1-GFP, was introduced to infect various cancer/non-cancer cell lines for screening and establishing an NDV-susceptible system. Six different human cell lines were used, including five cancer cell lines derived from various tissues, the colon (Caco-2), prostate adenocarcinoma cells derived from a metastatic site in bone (PC3), the lung (A549), the liver (Hep G2), the cervix (HeLa) and a non-cancerous fibroblast cell line developed from lung tissue (MRC5). All cell lines were infected with B1-GFP neat virus (MOI of 50) and its ten-fold dilutions. Time- and NDV dose-dependent cell viability was monitored during a nine-day period (Figure 1).

A consistent decrease in cell viability was only observed in the infected Caco-2 cells with all concentrations of virus (Figure 1A). The B1-GFP virus infection reduced cell viability by more than 90% within 48 h of neat virus infection. A gradual decrease in cell viability with more diluted virus was observed in successive 10-fold dilutions of the virus after 48 h post-infection in Caco-2 cells. PC3 (Figure 1B) and A549 (Figure 1C) cells were found to be moderately susceptible to the B1-GFP infection only at higher viral doses (neat virus, 10^−1^ and 10^−2^ dilutions for PC3 cells; neat virus and 10^−1^ dilution for A549 cells). Liver cancer Hep G2 cells were susceptible only to the B1-GFP neat virus infection within 72 h (Figure 1D), whereas HeLa cells remained unaffected by the viral infection regardless of viral dose or duration of the infection (Figure 1E). Within 48 h post-infection (neat virus and 1:10 dilution), the non-cancerous MRC5 cells showed a reduction in cell viability. However, with a 1:10 dilution of B1-GFP, an increase in the cell viability of MRC5 cells was observed after 5 days (Figure 1F).

Oncolytic viruses acquire oncotropism by favourably infecting and replicating in cancer cells, as cancer cells have deficient cell death pathways and weaker antiviral responses. On the contrary, non-cancer cells’ antiviral responses and cell death pathways are intact, hence non-cancer cells like MRC5 when infected with NDV at higher viral load are susceptible to death. However, with decreasing viral doses, MRC5 cells recovered from the infection and regained cell viability. The oncolytic efficacy of the virus is dependent on multiple factors, such as the efficiency of infection in cancer cells, type of virus, and natural and induced viral tropisms leading to the types of cell deaths, hence cancer cells like HeLa, Hep G2, PC3 and A549 cells showed varying degrees of response to NDV doses in a time-dependent manner.

### 3.2. Identification of Persistently Infected VR Caco-2 Cells

After examining the susceptibility of Caco-2 cells to B1-GFP virus, further study focused on the Caco-2 cells. With the neat virus, which resulted in an estimated MOI of 50, most cells were infected and consequently died after 48 h post-infection. A small number of surviving cells (<1%) were unintentionally found after 12 days under microscopic examination, despite a high level of oncolysis, named as virus-resistant Caco-2 (VR Caco-2) cells. The VR Caco-2 cells that initially survived required 4–6 weeks to form colonies and ~4 months to reach 80–90% confluence. Infected and mock-infected Caco-2 cells were observed routinely under a conventional inverted bright-field microscope to assess the cell morphology and cytopathic effects of NDV infection. Under the microscope, the VR Caco-2 cells proliferated much more slowly than the uninfected Caco-2 cells. 

**Figure 2:**
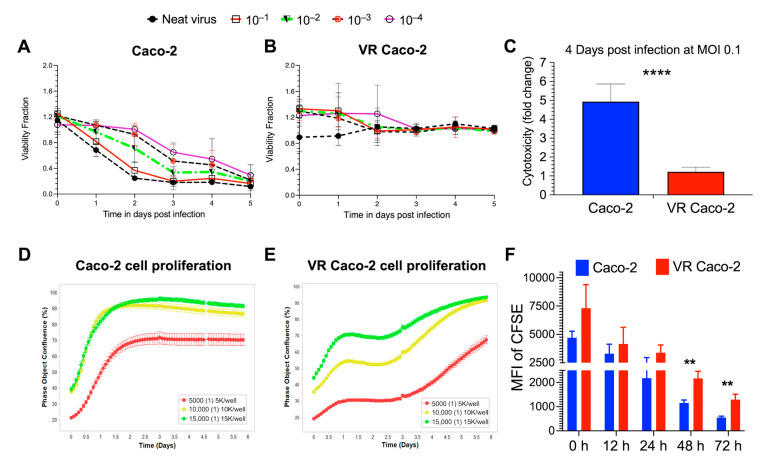
Characterisation of VR Caco-2 and Caco-2 cells. Cell viability measured in NDV B1-GFP neat virus and 10-fold virus dilutions infected (**A**) Caco-2 and (**B**) VR Caco-2 cells in a 5-day virus killing curve assay. NDV reinfection experiments demonstrated resistance in VR Caco-2 cells. (**C**) Cell cytotoxicity assay of 0.1-MOI NDV B1-GFP-infected Caco-2 and VR Caco-2 cells 4 days post-infection demonstrated reduced cytotoxicity of VR Caco-2 cells. Cell proliferation assays showing (**D**) Caco-2 and (**E**) VR Caco-2 cell confluence monitored in real time using the Incucyte imaging system at different seeding densities. Phase object confluence (%) represents the surface area covered by cell monolayer in the culture plate. (**F**) Mean fluorescence intensity (MFI) of CFSE-stained Caco-2 and VR Caco-2 cells at different proliferation time points. CFSE dilution is significant at 48 h and 72 h in Caco-2 cells, indicating greater numbers of cell divisions than VR Caco-2 cells. Data (mean ± SD) shown here are from three independent experiments and analysed using multiple non-parametric *t*-tests (****: *p* < 0.0001; **: *p* < 0.01).

NDV reinfection experiments with B1-GFP demonstrated a significant difference in the cell viability of the infected Caco-2 and VR Caco-2 cells (Figure 2A,B). While Caco-2 cells responded to B1-GFP infection in a dose-dependent manner (Figure 2A), no viral doses had an effect on VR Caco-2 cells at any time point, resulting in constant cell viability (Figure 2B). The cell cytotoxicity was measured in the infected Caco-2 and VR Caco-2 cells at low MOI of 0.1; Caco-2 cells showed significantly higher cytotoxicity than the VR Caco-2 cells upon infection, as illustrated in Figure 2C. Both tests confirmed the resistance of VR Caco-2 cells to NDV reinfection and NDV-induced oncolysis. 

The resistance to NDV superinfection and NDV-mediated cell death was acquired only in persistently infected (VR Caco-2) cells. VR Caco-2 cells produced a significantly low titre of replicating virus continuously. There are many possible reasons why only a small population of the cells sustained persistent infection. For instance, one possible explanation could be that Caco-2 is a heterogenous cell line known to differentiate spontaneously into different intestinal epithelial cells. Therefore, it is possible that some cells are more likely to inhabit persistent infection than others. The difference in the metabolic remodelling of parental wild-type Caco-2 cells relative to persistently NDV-infected VR Caco-2 cells is not substantial, as VR Caco-2 cells harbour a significantly low level of virus and do not become reinfected by NDV in the continuous cell culture. Although more research is required, it is possible that either receptor alteration of VR Caco-2 cells or the induction of an antiviral state in VR Caco-2 cells accounted for the resistance to reinfection.

Similar to the microscopic observation of slower VR Caco-2 cell growth compared to the uninfected Caco-2 cells, cell proliferation assays by real-time monitoring of the cell confluence confirmed the slower proliferation of VR Caco-2 cells (Figure 2D,E). Caco-2 cells entered the log phase after less than 12 h in the lag phase and reached more than 90% confluence in 1.5 days after being seeded at 10,000 and 15,000 cells/well, at which point they entered the stationary phase. A similar pattern was seen in 5000 cells/well that had reached 70% confluence and had begun stationary development (Figure 2D). Contrarily, VR Caco-2 cells entered the exponential phase after around three days in the lag phase across all three densities, regardless of confluence. At all cell densities, VR Caco-2 cells displayed a distinct growth phase curve from Caco-2 cells. It took VR Caco-2 cells 5.5 days to reach >90% cell confluence at seeding densities of 10,000 and 15,000 cells/well (Figure 2E). The CFSE cell proliferation experiment confirmed that the growth of VR Caco-2 cells was twice as slow as that of Caco-2 cells at 48 and 72 h after CFSE labelling (Figure 2F). Notably, VR Caco-2 cells displayed similar morphology in the cell culture monolayers as the Caco-2 cells.

### 3.3. Persistent VR Caco-2 Can Be Identified by Metabolic Profiles at Single-Cell Level

**Figure 3:**
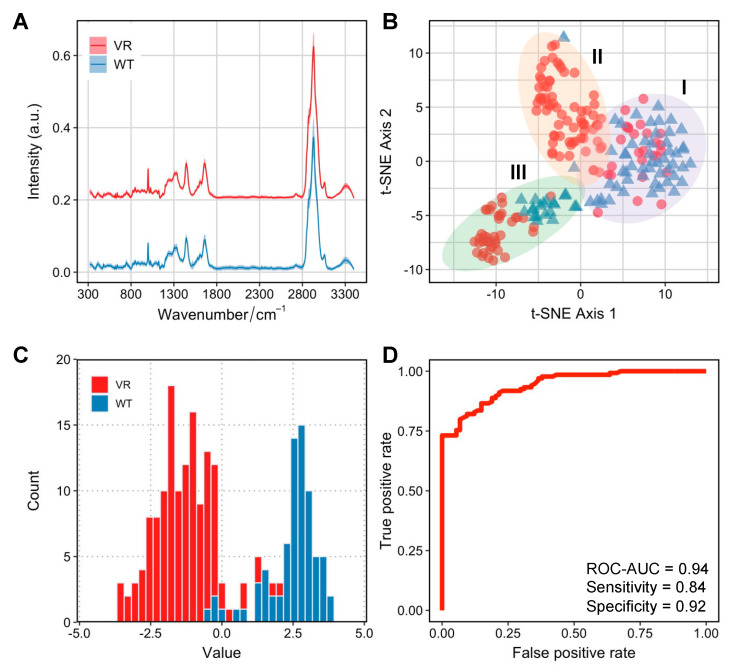
Distinct Raman profiles of Caco-2 and VR Caco-2 cells. (**A**) Raman spectra of VR Caco-2 and Caco-2 cells averaged from 218 and 92 single cells, respectively. The shaded area represents standard deviation at each wavenumber from single-cell measurements. Raman spectra from each group was shifted and scaled in intensity to aid visualisation and the intensity is expressed in arbitrary unit (a.u.). (**B**) Unsupervised tSNE visualisation observes three distinct clusters I, II and III comprised of heterogeneous Caco-2 and VR Caco-2 single cells. (**C**) Histogram of values of a linear discriminant analysis (LDA) model to differentiate Caco-2 and VR Caco-2 based on their Raman spectra. (**D**) A receiver operating characteristic (ROC) curve was used to evaluate the LDA model performance. The area under the ROC curve (ROC-AUC) was 0.94 where 1 indicates perfect classification.

Despite initially being the most susceptible cell line for NDV-induced oncolysis, Caco-2 cells developed infection persistence in a small population of VR Caco-2 cells. To understand whether the metabolic profiles can be discriminated in the two groups of cells responsible for distinct infection manifestations, we employed single-cell Raman spectroscopy to investigate Caco-2 and VR Caco-2 cells. The averaged Raman spectra for VR Caco-2 and Caco-2 cells are shown in Figure 3A, averaged from 218 and 92 single cells, respectively. The fingerprint region (300–1800 cm^−1^) illustrates biomolecular vibrational modes within a single cell, and the high wavenumber region (2800–3100 cm^−1^) shows the intensive C–H stretching vibrations from lipids, proteins and nucleic acids.

First, an unsupervised t-distributed stochastic neighbour embedding (tSNE) analysis was used to visualise the high-dimensional Raman dataset in a two-dimensional space (Figure 3C). Interestingly, a degree of heterogeneity within populations was observed, especially in the VR Caco-2 cells. Three clusters were identified via k-means clustering. Cluster I was comprised mostly of the wild-type Caco-2 cells (73%); some of the VR Caco-2 cells in this cluster might still have had phenotypic resemblance to the wild-type cells. Cluster II was mostly made up of VR Caco-2 cells (97%) while cluster III, the most distant from the other two clusters, was the most heterogeneous (34% Caco-2 wild type and 66% VR Caco-2). 

We then visualised the Raman profiles of the two groups in a supervised manner via linear discriminant analysis (LDA). The histogram of the LD values from the LDA clearly separates the VR Caco-2 and Caco-2 cells (Figure 3C). In a binary classification task using LDA for differentiating VR Caco-2 and Caco-2 cells based on their SCRS, a receiver operating characteristic (ROC) curve was used to evaluate the model performance (Figure 3D). The area under the ROC curve (ROC-AUC) achieved 0.94 at single-cell level, where 1 indicates perfect classification. The supervised machine learning model demonstrates that the Raman profiles of the VR Caco-2 cells are distinctly different from those of the uninfected parental Caco-2 cells, suggesting significant metabolic reprogramming in those persistently infected cells. The development of persistent infections for oncolytic viruses may result in reduced oncolytic potential and compromised therapeutic outcomes [15]. Importantly, the persistent cancer cells are able to proliferate and produce populations that are resistant to the viruses. The ability to identify the subpopulations of resistant cells from the wild-type cancer cells is imperative in clinical settings. 

### 3.4. Raman Profiles Differentiate VR Caco-2 and Caco-2 Cells

**Figure 4:**
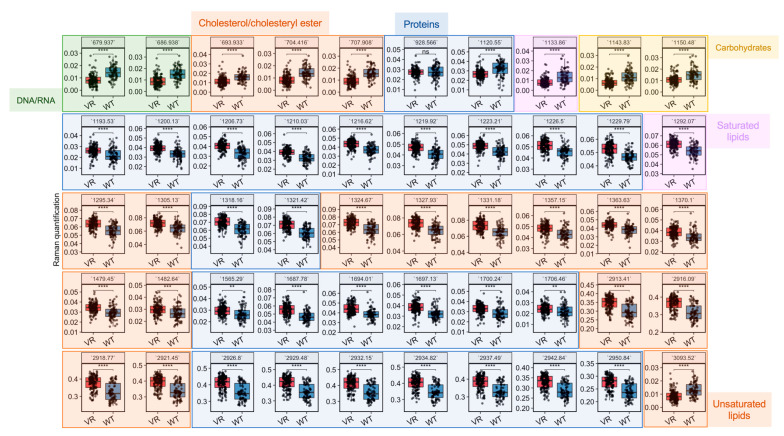
Quantification of biomolecules in Caco-2 and VR Caco-2 based on their SCRS. Differential Raman bands selected based on the LDA model were quantified and compared between Caco-2 and VR Caco-2 cells. All Raman bands are colour-coded as green (DNA/RNA), orange (lipids) or blue (proteins). Statistics was done with Student’s *t*-test (ns: *p* > 0.05; **: *p* < 0.01; ***: *p* < 0.001; ****: *p* < 0.0001). VR: VR Caco-2; WT: Caco-2.

Based on the most important variables selected by the LDA model (Figure S1), differential biomolecules were semi-quantified by integrating corresponding Raman bands and compared between VR Caco-2 and Caco-2 cells (Figure 4 and Table 1). Assignment of a Raman band is most accurate for the corresponding vibrational modes; nevertheless, assignments to biomolecules with specific vibrations are possible. A number of changes related to DNA/RNA, lipids and proteins are colour-coded in green, orange and blue, respectively, in Figure 4. Raman bands related to DNA/RNA at 680 cm^−1^, which can be assigned to ring breathing modes in the DNA bases, were significantly lower in the resistant cells compared to the uninfected parental cells. This is consistent with the observation in the cell proliferation experiments that VR Caco-2 cells had a much slower replication rate than Caco-2 cells. A number of Raman biomarkers related to protein synthesis were significantly lower in the VR Caco-2 cells, including Amide III at 1200–1300 cm^−1^, Amide I at 1600–1690 cm^−1^ and C–C vibrations of tryptophan and phenylalanine at 1210 cm^−1^ (Figure 4). An increase in protein synthesis and a decrease in nucleic acids in the VR Caco-2 cells suggest that energy could be redirected from proliferation to anabolism in those resistant cells for synthesising more proteins and metabolic reprogramming.

The lipidic profiles were also significantly modified in the VR Caco-2 cells. Several bands of saturated lipids exhibited an increase in the VR Caco-2 cells; for example, 1295 cm^−1^ for CH_2_ deformation, 1370 cm^−1^ for CH_3_ stretching, 1480 cm^−1^ for CH_2_ bending and 2913 cm^−1^ for CH_2_ stretching. On the other hand, =CH stretching for unsaturated lipids and vibrations in cholesterols and cholesteryl esters demonstrated significant decreases in the VR Caco-2 cells compared to the parental Caco-2. Production of phospholipids in combination with deceased proliferation rates suggests the development of a more saturated and robust plasma membrane. A plasma membrane is made up of lipids, particularly phospholipids and cholesterol, which function as integral pump proteins for multidrug efflux transporters from the ATP-binding cassette (ABC) superfamily. It has been shown that efflux pump activity could be modulated via the development of a more robust plasma membrane for favourable drug binding and release, thus conferring resistance to anti-cancer agents [1]. A decrease in the C–C and C–O ring breathing in the carbohydrates at 1150 cm^−1^ was also observed, suggesting that utilisation of carbohydrates might not be advantageous and the resistant Caco-2 cells might use alternative nutrients, such as lipids.

### 3.5. Raman-DIP Reveals Metabolic Reprogramming via Newly Synthesised Biomolecules

**Figure 5:**
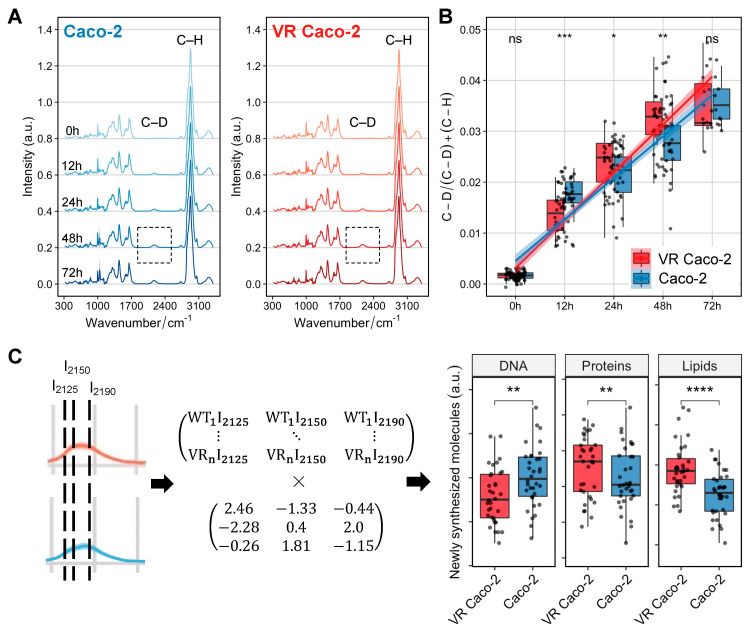
Raman-DIP of newly synthesised DNA, proteins and lipids. (**A**) Raman spectra of VR Caco-2 and Caco-2 cells cultured with 30% D_2_O at different incubation time points. The Raman band of C–H vibrations was shifted to a C–D band at 2070–2300 cm^−1^, highlighted in the grey box. (**B**) Metabolic activities of VR Caco-2 and Caco-2 cells were quantified and compared at each time point by calculating the ratio of (C–D) / (C–D) + (C–H). (**C**) Linear unmixing algorithm for calculating newly synthesised DNA at 2125 cm^−1^, proteins at 2150 cm^−1^ and lipids at 2190 cm^−1^. WT: Caco-2; VR: VR Caco-2. Statistics was done with Student’s *t*-test (ns: *p* > 0.05; *: *p* < 0.05; **: *p* < 0.01; ***: *p* < 0.001; ****: *p* < 0.0001).

We next sought to use Raman-DIP to probe the general and specific metabolic activities of VR Caco-2 and Caco-2 cells. SCRS of cells incubated with D_2_O showed a distinguishable C–D band centred at 2170 cm^−1^, with its intensity gradually increased from 0 h to 72 h in both VR Caco-2 and Caco-2 cells (Figure 5A). This band was shifted from the C–H stretching from 2800–3100 cm^−1^ due to the incorporation of D via C/D exchange during active metabolism. Hence, the D incorporation in cells can be used as a universal indicator of metabolic activity and was calculated as the ratio of (C–D) / (C–D) + (C–H).

Figure 5B shows the comparison of D incorporation, hence the metabolic activities, between the two groups of cells at 0, 12, 48 and 72 h. At 12 h, Caco-2 cells exhibited a higher metabolic rate compared to the VR Caco-2 cells, in agreement with the cell proliferation assay showing that the Caco-2 cells replicated much faster than the VR Caco-2 cells and had a much shorter lag phase (Figure 2D,E). Intriguingly, metabolism at 24 and 48 h suggested opposite results to the proliferation assay. Despite being two times slower in proliferation at 48 h and 72 h (Figure 2F), VR Caco-2 showed a significantly higher metabolic activity at 24 and 48 h (Figure 5B). At 72 h, the Caco-2 cells caught up and the 2 groups of cells had similar metabolic activities.

Due to the statistical significance at 48 h in both the proliferation assay and DIP experiment, the C–D band at 48 h was selected for further analysis. Previous studies have shown the C–H signal is a linear overlap of C–H stretching from lipids, proteins and DNA; therefore, the C–D signal is a combination of de novo lipid synthesis (I_2190_), de novo protein synthesis (I_2150_) and DNA replication (I_2125_) [16,17]. The C–D band can therefore be resolved into individual components of newly synthesised biomolecules via a linear unmixing algorithm (Figure 5C). At 48 h, both the de novo protein and de novo lipid synthesis in the VR Caco-2 cells increased, while the newly synthesised DNA was significantly lower than the control Caco-2 cells. Together with the decreased DNA/RNA content and increased protein synthesis (Figure 4), the DIP experiments suggest that the escape strategy of the resistant VR Caco-2 cells from the viral infection might be slowing down their replication and redirecting the energy to protein synthesis and metabolic reprogramming, such as forming a more robust plasma membrane. Notably, this finding should be tested in other cell lines to determine if it is one of the universal mechanisms to generate resistance. In future studies, it should also be confirmed using other technologies, such as measurements of cellular respiration or lipidomic mass spectroscopy.

Resistance to chemotherapy and targeted small molecule inhibitors has been found in multiple cancer types [18,19,20,21]. It has been proposed that a sizeable proportion of “idling” cells contribute to the persistent disease in BRAF-mutated melanoma patients after targeted inhibitor therapy [18]. Persisters’ observed nonquiescent and antiproliferative properties matched well with our persisters’ slower proliferation but more active metabolism. It has also been found that upregulation of antioxidant genes and metabolic engineering towards fatty acid oxidation are associated with persisters across multiple cancer types [22], which may correlate with the observation of increased fatty acid synthesis in VR Caco-2 cells. Our study provides phenotypic and metabolic evidence of cancer cells’ resistance to viral infection, while little is known about the mechanisms of resistance to viral therapy. Moreover, the emerging reservoir of heterogeneous drug-resistance cells and mechanisms [20] highlights the importance of single-cell techniques in investigating drug-tolerant cancer persister cells. By combining the power of optical magnification and confocality, Raman micro-spectroscopy even has the potential to explore biological processes at subcellular level. It may collectively provide label-free Raman images with subcellular structural and chemical information. In future studies, spatially resolved Raman images could be generated to locate biomolecules within a specific cellular compartment and therefore further pin down the underlying mechanistic pathways.

**Table 1:** Molecular vibrations and biological assignment of Raman wavenumbers of importance via LDA model.

Raman Wavenumber (cm^−1^)	Molecular Vibrations and Biological Assignment	Reference
680	Guanine; ring breathing modes in the DNA bases/C-2′-endo-anti	[23]
700	Cholesterol, cholesterol ester	[24]
929	Proline, hydroxyproline, ν(C–C) skeletal of collagen backbone	[25]
1121	C–N stretching of proteins	[26]
1134	ν(C–C) skeletal of acyl backbone in lipid (trans conformation)	[27]
1150	C–C, C–O ring breath, glycogen	[28]
1200	Amide III and CH_2_ wagging vibrations	[29]
1210	Tryptophan and phenylalanine ν(C–C6H5) mode	[30]
1230–1300	Amide III (arising from coupling of C–N stretching and N–H bonding)	[26]
1295	CH_2_ deformation in lipids	[24]
1320	Amide III, C–H deformation	[31]
1370	CH_3_ stretching in phospholipids	[32]
1479	CH_2_ bending mode of proteins and lipids	[33]
1483	CH_2_ bending mode of proteins and lipids	[33]
1480–1580	Amide II of proteins	[26]
1600–1690	Amide I of proteins	[26]
2913	CH_3_ stretching of lipids	[24]
2950	CH_3_ stretching of proteins	[23]
3010	Unsaturated =CH stretching	[24]

## 4. Conclusions

Resistance of cancer cells to NDV is a major obstacle to the development of the oncolytic virus as a promising cancer treatment. Upon infection of a non-pathogenic strain of NDV to a range of cancer cell lines, Caco-2 cells were found to be most susceptible to NDV killing. However, a subpopulation of Caco-2 cells with persistent infection was identified as VR Caco-2. Compared to their non-infected counterpart, VR Caco-2 cells grew much more slowly and demonstrated resistance towards NDV reinfection and NDV-induced cell death. To understand the metabolic adaptations of the resistant VR Caco-2 cells, single-cell Raman spectroscopy and Raman-DIP techniques were employed to identify the persistent NDV infection in cancer cells. The LDA model has achieved high performance in distinguishing VR Caco-2 and Caco-2 cells at single-cell level with an ROC-AUC of 0.94. Furthermore, the metabolism of those resistant cells was investigated. The Raman profiling and Raman-DIP experiments unveiled an increased de novo synthesis of proteins and lipids, accompanied by a decreased DNA/RNA synthesis. Despite having a much slower replication rate, the VR Caco-2 cells exhibited more active metabolism at 24 and 48 h after infection. The results suggest that the resistant VR Caco-2 cells might employ an escape mechanism from the viral infection to slow down their replication and redirect the energy to protein synthesis and metabolic reprogramming. 

As with other cancer therapies, treatments based on oncolytic viruses like NDV face the challenge of resistance. The existence of a subpopulation of drug-resistant cancer cells poses difficulty and complexity in identifying them due to their small number, <1% in the case of Caco-2. Being able to recognise them at single-cell level with high accuracy would be hugely beneficial in the process of developing new cancer treatment. Applying the methods in combinational and personalised medicine, Raman and Raman-D_2_O techniques can quickly identify and characterise resistant cells that are still metabolically active based solely on their metabolic phenotypes, thus helping equip better strategies and improve efficacy for fighting treatment resistance in cancer.

## Data Availability

Data is available from Figshare at 10.6084/m9.figshare.21960743.

## Supplementary Materials

The following supporting information can be downloaded at: https://www.mdpi.com/article/10.3390/cancers15030811/s1, Figure S1: Variable importance of Raman wavenumber contributing to the LDA classification.
