# Supplementary material for: Metabolic Reprogramming in Colon Cancer Cells Persistently Infected with Newcastle Disease Virus
Source: Cancers (Basel). 2023 Jan 28;15(3):811. doi: 10.3390/cancers15030811 (PMC9913782; doi:10.3390/cancers15030811)
Supplement: Supplementary file 1 [file cancers-15-00811-s001.zip › cancers-2089706-supplementary.pdf]

Metabolic reprogramming in persistent colon cancer cells infected by Newcastle disease virus

Tong Yu[1,#], Archana Chandrabhan Jadhav[2,3,#], Jiabao Xu[1,*], Adrian L. Harris[4], Venugopal Nair[3], Wei E. Huang[1,*]

[1]Department of Engineering Science, University of Oxford. Parks Road, Oxford OX1 3PJ, UK.

[2]Beamline B24, Diamond Light Source, Harwell Science and Innovation Campus, Didcot, Oxfordshire, OX11 0DE, UK

[3]Viral Oncogenesis Group, The Pirbright Institute, Pirbright, Woking, Surrey, GU24 0NF, UK & Department of Biology, University of Oxford, 11a Mansfield Road, Oxford OX1 3SZ, UK

[4]Molecular Oncology Laboratories, Department of Oncology, Weatherall Institute of Molecular Medicine, John Radcliffe Hospital, Oxford University, Oxford OX3 9DS, UK

[#]The authors contributed equally to this work.

*Correspondence: Jiabao Xu, jiabao.xu@eng.ox.ac.uk; Wei E Huang, wei.huang@eng.ox.ac.uk

This PDF file includes: Figure S1

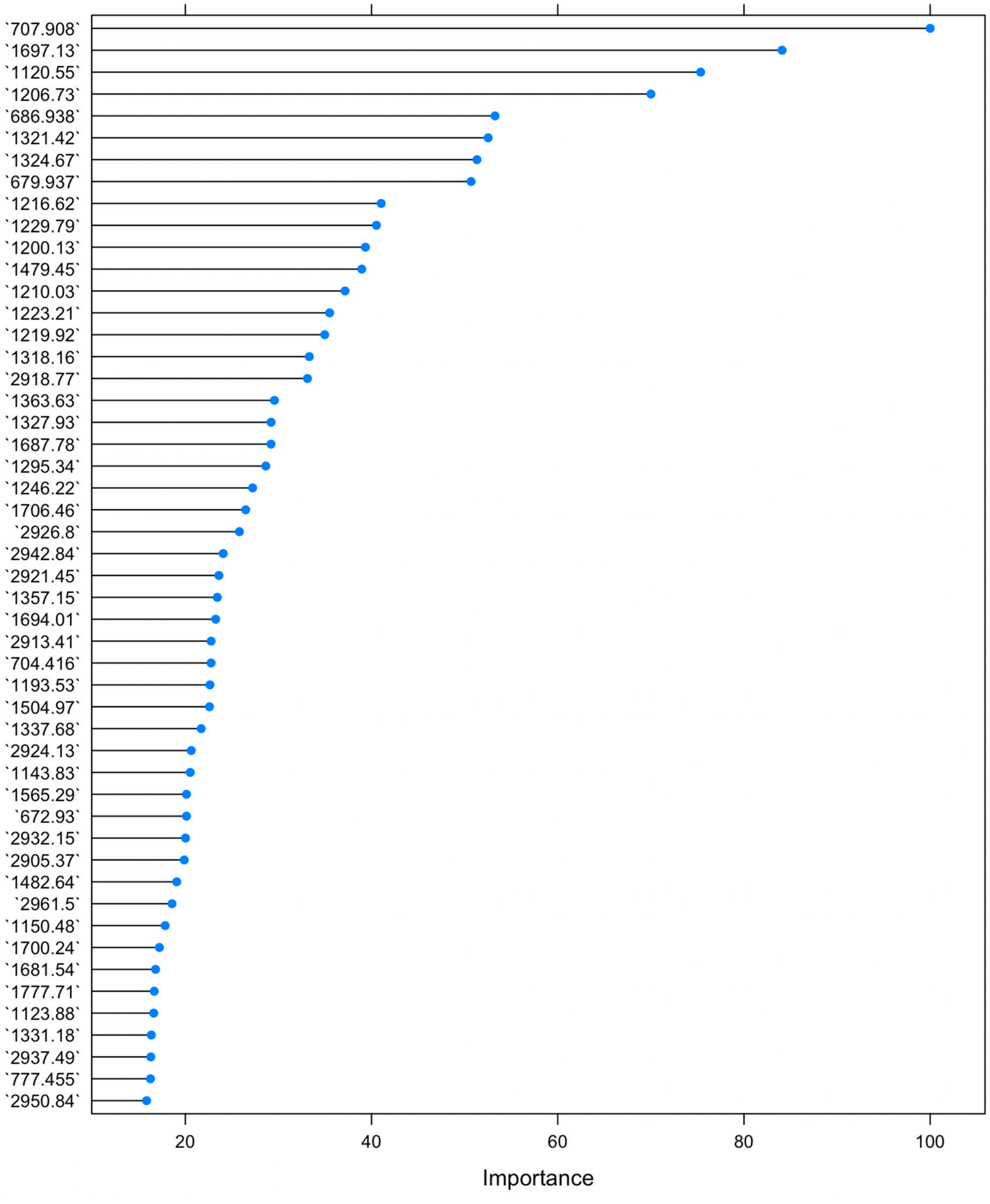

Figure S1. Variable importance of Raman wavenumber contributing to the LDA classification.
